# Growth Performance and Carcass Quality in Broiler Chickens Fed on Legume Seeds and Rapeseed Meal

**DOI:** 10.3390/ani10050846

**Published:** 2020-05-14

**Authors:** Jakub Biesek, Joanna Kuźniacka, Mirosław Banaszak, Sebastian Kaczmarek, Marek Adamski, Andrzej Rutkowski, Anna Zmudzińska, Katarzyna Perz, Marcin Hejdysz

**Affiliations:** 1 Department of Animal Breeding, Faculty of Animal Breeding and Biology, UTP University of Science and Technology in Bydgoszcz, Mazowiecka 28, 85-084 Bydgoszcz, Poland; jakub.biesek@utp.edu.pl (J.B.); kuzniacka@utp.edu.pl (J.K.); miroslaw.banaszak@utp.edu.pl (M.B.); adamski@utp.edu.pl (M.A.); zmudzinska@utp.edu.pl (A.Z.); 2 Department of Animal Nutrition and Feed Management, UP Poznań University of Life Sciences, Wołyńska 33, 60-637 Poznań, Poland; sebastian.kaczmerek@up.poznan.pl (S.K.); anrut@up.poznan.pl (A.R.); 3 Department of Animal Breeding and Product Quality Assessment, UP Poznań University of Life Sciences, Wołyńska 33, 60-637 Poznań, Poland; katarzyna.perz@up.poznan.pl

**Keywords:** broiler, rearing, legumes, protein, quality of meat, content of muscles, fatness

## Abstract

**Simple Summary:**

The production of broiler chickens is a dynamically developing industry. Most feeds are based on soybean meal as a source of protein. However, new challenges are arising in the meat production market, and consumers expect a wider range of products to choose from. There is potential in the production of broiler chickens fed on other sources of protein, e.g., legume seeds and rapeseed meal. The aim of this study was to investigate whether other sources of protein give similar results in terms of growth performance and meat quality compared to soybean meal diets. It was shown that the use of legume seeds and rapeseed meal did not affect most of the carcass characteristics and meat quality. However, the growth performance results were lower than when using soybean meal. Because they contained the smallest amount of antinutritional substances, faba beans are proposed as a possible partial replacement for soybean meal.

**Abstract:**

The aim of this study was to compare the growth performance parameters, carcass quality, and meat traits in broiler chickens fed on diets containing legume seeds and rapeseed meal as an alternative to soybean meal. In this study, 448 male ROSS 308 chicks were divided into subgroups: a control group (I) fed on soybean meal (SBM), and six experimental groups II—rapeseed meal (RSM); III—white lupin (WY); IV—yellow lupin (YL); V—narrow-leaved lupin NLL; VI—pea (Pe); and VII—faba bean (FB). After 42 days of rearing, 10 birds from each group were slaughtered and dissected. The control group was characterized by better growth performance compared to the other groups. In addition, the European Broiler Index was lower in each experimental group compared to the SBM group. A lower dressing percentage was found only in the NLL group. The muscle content in birds from the RSM and FB groups was significantly higher than in the other groups, but the fat content was lower. Meat from SBM group was characterized by the highest protein content, but a reduced content of fat and water in the muscles. The most similar results were found between the control group and the FB group receiving a diet based on faba beans. Furthermore, lupins had a similar effect on the carcass traits when used in the diets. The quality of meat in broilers fed on faba beans with the addition of potato protein and brewers’ yeast was similar to that of those fed on soybean meal, because the antinutrients were the lowest in faba bean seeds. Faba beans are proposed as a possible alternative source of protein in poultry diets. Other legume seeds should be analyzed in future studies.

## 1. Introduction

Global poultry production has been growing dynamically. In 2017, global poultry meat production was over 117.7 million tons, representing an increase of 0.4% compared to 2016 [1]. Poultry meat is characterized by its good quality, high level of safety, affordable prices compared to other meat types, and short cycles of production [2]. A growing demand for poultry meat and increased production volume, as well as increased exports and imports, have been observed [3].

Diet is one of the key factors affecting the quantity and quality of poultry meat and the profitability of production. Feed supplied to birds not only affects their growth rate, feed intake, and feed conversion ratio per kg of body weight gain, but also the water intake, hydration of excreta, and the quality of litter. The quality of the carcass and meat also depend on the genotype, sex, and age of birds, and their management system [4,5,6,7,8]. Genetically modified soybean meal (SBM) is a popular dietary ingredient for formulating poultry feed mixtures. Birds fed on SBM achieve the best growth performance [9]. However, because Polish legume cultivars have shown potential in poultry feeding with their high protein content, the search for alternative sources of protein has continued for years. This trend is also driven by the need to secure the supply of protein, because Poland imports about 2–3 million tons of soybean meal to satisfy the demand for protein-rich feed for animals. High levels of protein are found, for example, in legume seeds, which are increasingly being used in the diets of poultry and other livestock [10,11,12,13,14].

Currently, new varieties of legumes contain low levels of antinutrients, which makes them candidates for use as high-protein components in feed for poultry. The impact of the use of legume seeds in feed on the quality of meat of broiler chickens has been the subject of research by other authors. Hejdysz et al. [12] found no negative effects associated with the use of faba beans (raw and extruded) on chicken meat quality. Similarly, Kuźniacka et al. [15] reported that the use of faba beans in broilers’ diets had no significant effect on most meat quality parameters, with a slight effect on fatty acid composition and collagen content in the breast muscles. In turn, Kiczorowska et al. [16] found that the use of micronized pea seeds instead of soybean meal had a positive effect on the composition of fatty acids and on the atherogenic and thrombogenic indices in chicken breast muscles. Partial replacement of soybean meal with peas (200 g/kg) in chicken nutrition has been shown to improve production results and carcass traits [17]. Research in this area was also conducted on other bird species. Kuźniacka et al. [18] used different sources of protein (yellow lupin, rapeseed meal, narrow-leaved lupin, pea) in various combinations as replacements for soybean meal in the feeding of ducks. A similar quality of meat was found in ducks fed on yellow lupin and rapeseed meal compared to ducks fed on soybean meal. Similarly, Tufarelli and Laudadio [19] showed an improvement in the production results and carcass traits of guinea fowl, where the feed was based on dehulled micronized faba beans. As previous studies indicate, legumes as replacements for soybean meal do not have a negative impact on the quality of poultry meat, which indicates the validity of conducting research and finding the best species of legume for use as a high-protein feed component in poultry nutrition.

The tested hypothesis: Various legume seeds and rapeseed meal in diets affect the growth performance and the quality of meat of broiler chickens.

The aim of the study was to compare the growth performance parameters, carcass quality, and meat traits in broiler chickens fed on diets containing legume seeds and rapeseed meal as an alternative to soybean meal.

## 2. Material and Methods

Meat quality research does not require the consent of an ethical committee, and the slaughter was carried out in a professional slaughterhouse. The experiment was treated as a routine production cycle in small-scale conditions. Our research aim was mainly to analyze the quality of the carcasses and meat (directive No. 2010/63/EU).

### 2.1. Legume Seeds

Yellow lupin (*Lupinus luteus* cv. Mister), narrow-leaved lupin (*Lupinus angustifolius* cv. Sonet), white lupin (*Lupinus albus* cv. Butan), and pea seeds (*Pisum sativum* cv. Tarchalska) were obtained from plant breeding stations (Wiatrowo, Poland). Faba beans (*Vicia faba* cv. Olga) were obtained from plant breeding stations in Strzelce, Łódzkie Voivodeship, Poland. Before experimental diet preparation, seeds were ground using a model RG11 hammer mill (Zuptor, Gostyń, Poland) with a screen size of 2.0 mm. The chemical compositions of the legume seeds are presented in Table 1.

### 2.2. Animals and Diets

The study was conducted on 448 1 day old male ROSS 308 chicks. Before the experiment, all birds were vaccinated in the hatchery. The birds were randomly allocated to the control group (I) or one of six experimental groups (II–VII). Each group was divided into eight replicates, with eight birds per replicate. Birds were reared on a wood-shaving litter in 1.2 × 0.8 m pens with a semi-automatic feeder and five droplet drinkers. Birds were exposed to the light for 24 h/day in the first 7 days, followed by 18 h light–6 h darkness (28 days). The temperature was maintained at 32 °C during the first week and was gradually decreased to approximately 23 °C by the end of the experiment. Feed and water were available ad libitum. The diets were isoenergetic and isonitrogenic. In order to equalize the protein level in all experimental diets, potato protein and brewers’ yeast were added in an amount that would not decrease the broiler chickens’ performance [20]. The protein sources alternative to SBM used in each group were: II—rapeseed meal (RSM), III—white lupin (WL), IV—yellow lupin (YL), V—narrow-leaved lupin (NLL), VI—pea (Pe), and VII—faba bean (FB). In each group, the content of legumes was 25%. Chickens were reared to the age of 42 days. The birds were fed on starter mixtures between Days 1 and 14 (Table 2), and then on grower mixtures between Days 15 and 42 (Table 3). All experimental diets were provided in pellet form and were formulated based on the analyzed chemical composition of all diet components and the nutritive values of feedstuffs published by Smulikowska and Rutkowski [21]. Chickens and feed were weighed twice during the rearing period (on Days 14 and 42 of the experiment). In the experiment, the growth performance parameters were evaluated: body weight gain (BWG), feed intake (FI), and feed conversion ratio (FCR) for three feeding phases (Days 1–14, Days 15–42, and Days 1–42). Additionally, the European Broiler Index (EBI) was calculated using the formula (average grams gained/day X% survival rate)/ feed conversion × 10. After 42 days of rearing, 10 birds from each group (one bird/replication and additionally two birds approximately equal to the average mass of the group) were anesthetized by CO_2_ asphyxiation, slaughtered by cervical dislocation, and dissected.

### 2.3. Meat Quality

The pH value of the breast muscles was measured 15 min post-mortem (pH_15_) using a CX-701 pH-meter with a knife electrode from Elmetron (Poland). The post-mortem parameters, including carcass weight, weight and proportion of individual carcass elements, and dressing percentage were analyzed [22]. The pH value of breast muscles was also measured 24 h post-mortem (pH_24_). The color of the breast and leg muscles was assessed using a colorimeter (Konica Minolta, model CR400, Tokyo, Japan), calibrated using the white calibration plate no. 21033065 and the D_65_ Y_86 1_ x_0.3188_ y_0.3362_ scale. We graded color according to the CIE L*a*b* system (L* = lightness, a* = redness, and b* = yellowness) [23], analyzed the water-holding capacity with the Grau and Hamm method [24], and measured the drip loss [25]. We also measured the content of protein, fat, water, and collagen in the meat [26] with a Near Infrared Transmission (NIT) spectrometer calibrated for artificial neural network (ANN) with a FoodScan apparatus from FOSS (Denmark).

### 2.4. Analytical Methods

For chemical analyses, representative samples of seeds were ground to pass through a 0.5 mm sieve. Soybean and rapeseed meal as well as legume seeds were analyzed in duplicate for crude protein (CP) and ether extract (EE) using Methods 976.05 and 920.39, respectively, according to the Association of Official Analytical Chemists (AOAC) procedures [27]. Additionally, acid detergent fiber (ADF), expressed inclusive of residual ash and neutral detergent fiber (NDF), with heat-stable amylase, was analyzed in the seeds (Procedures 942.05 and 973.18, respectively, according to the AOAC, [27]). The starch content in pea and faba bean seeds was determined using a diagnostic assay kit for agricultural industries (Megazyme International; AOAC, 2005: Method 996.11, Dublin, Ireland) based on the use of thermostable α-amylase and amyloglucosidase. The AA content was determined on an AAA-400 Automatic Amino Acid Analyzer (INGOS s.r.o., Prague, Czech Republic), using ninhydrin for postcolumn derivatization (procedure 994.12; AOAC, [27]). In the pea and faba bean samples, the tannin content was analyzed according to the method of Kuhla and Ebmeier [28]. The raffinose-family oligosaccharides (RFO) were extracted and analyzed by high-resolution gas chromatography, as presented by Zalewski et al. [29]. Phytate was determined according to the method of Haug and Lantzsch [30]. Lupin alkaloids were extracted from the flour by trichloroacetic acid and methylene chloride (Sigma-Aldrich, Munich, Germany). The determination of alkaloids was carried out via gas chromatography (GC) (Shimadzu GC17A, Kyoto, Japan) using a capillary column (Phenomenex, Torrance, CA, USA).

### 2.5. Statistical Analysis

Numerical data were analyzed using statistical software (SAS, 2012). A one-way analysis of variance (ANOVA) was used. The significance of the differences between the mean value of SBM group and mean values of the other groups was estimated using Dunnett tests, at a significance level of *p* ≤ 0.05.

## 3. Results

### 3.1. Chemical Composition of Legumes

According to Table 1, the highest level of crude protein (CP) was found in soybean meal seeds (451 g/kg dry matter (DM)) and yellow lupin seeds (425 g/kg·DM), while pea seeds had the lowest level of CP (204 g/kg·DM)). The lowest content of acid detergent fiber (ADF) and neutral detergent fiber (NDF) was found in the soybean meal (77 g/kg·DM; 119 g/kg·DM, respectively) and a low level of ADF was found in the pea seeds (91 g/kg·DM). The seeds showed the highest levels of ADF for narrow-leaved and white lupin (205; 210 g/kg DM, respectively), and of NDF in yellow and narrow-leaved lupin (252; 255 g/kg·DM, respectively). The starch content was determined only in the pea and faba bean seeds at a level of 590–480 g/kg·DM, while in the other legume seeds, the level of starch was not determined, because they contained a very low content (about 1%). Only in each lupin cultivar was the total alkaloid content found at the level of 0.0009–0.0019 g/kg·DM, while peas and faba beans were characterized by the presence of tannins (0.02–0.06 g/kg·DM), with the lowest level of phytin phosphorus (p-phyt., 1.7–1.8 g/kg·DM). The highest levels of oligosaccharides and raffinose were found in yellow lupin seeds (134.27; 10.21 g/kg·DM, respectively), and the lowest in rapeseed meal seeds (20.4; 3.4 g/kg·DM, respectively), in which the highest level of p-phyt was found (7.9 g/kg·DM).

### 3.2. Chickens’ Performance

Mortality was negligible and only 1% of birds died. Birds from the control group, where the feed was based on soybean meal, were compared to the experimental groups characterized by the highest results of growth performance parameters. Body weight gain (BWG) (Table 4) was significantly higher in each feeding phase (Days 1–14, Days 15–42, Days 1–42) in the SBM group (soybean meal) than in the experimental groups. In the first phase of feeding, only the Pe group fed with pea seeds was characterized by a significantly lower feed intake than the control group fed with soybean meal (*p* = 0.003). However, in the second phase and over the total rearing period, a significantly higher feed intake in groups fed with white lupin (WL), yellow lupin (YL), and narrow-leafed lupin (NLL) was noticed in comparison to the SBM group (*p* < 0.001). The FCR parameter was significantly higher in every experimental group in comparison to the control group fed with soybean meal (*p* < 0.001) during the whole rearing period and in each feeding phase. Additionally, the European Broiler Index (EBI) was also the highest in the control group (SBM) compared to the rest of the groups, which were fed with rapeseed meal and various legume seeds (*p* < 0.001).

### 3.3. Meat Traits

Significant differences were found in the pre-slaughter body weight parameter in groups fed with RSM, NLL, and Pe as compared to the SBM group. The experimental groups, characterized by lower body weight gain than the SBM group, had a mean value of 2370 g. After slaughter, the gutted carcasses with necks of birds fed with various lupins (white, yellow, and narrow-leafed lupins), as well as with pea seeds, had lower values than the birds from the SBM group, with a mean carcass weight of 1680 g (*p* < 0.001). A significantly lower dressing percentage (66.53%) and a higher weight and proportion of neck was noticed only in the NLL group, compared to the SBM group (*p* < 0.001), with values of 65.70 g, 4.47% (NNL), and 36.10 g; 2.15% (SBM), respectively. In the rest of the traits, no significant differences were found (*p* > 0.05) (Table 5).

As Table 6 shows, a significantly lower weight of breast muscles was found in the RSM, WL, YL, and Pe groups (*p* < 0.001) with values between 306.9 and 367.9 g, as compared with the SBM group (440.1 g). At the same time, the proportion of breast muscles in the carcasses was significantly lower in the YL group only, as compared with the SBM group (*p* < 0.001). Similarly, in the RSM and Pe groups, leg muscles and their proportion in the carcass was significantly lower (*p* < 0.001). The same results were noticed in the NLL group (*p* < 0.001). However, the total muscle weight and proportion of the Group I carcass results were a little bit different from those of the divided muscles. Birds fed with soybean meal (SBM group) were characterized by significantly higher results regarding the aforementioned traits as compared with the RSM, YL, NLL, and Pe groups (*p* < 0.001). Each experimental group, excluding the Pe group, was characterized by a lower weight of skin including the subcutaneous layer (*p* < 0.001), but only birds fed with white and narrow-leafed lupin seeds (WL, NLL) had a significantly lower proportion of skin including the subcutaneous layer as compared with the SBM group (*p* < 0.001). Birds fed with faba beans (FB) had a much lower proportion of abdominal fat (8.90 g) than the SBM group (16.70 g), which was statistically significant (*p* < 0.001). However, the proportion of abdominal fat in the carcasses was significantly higher in the RSM group (1.55%) than in the SBM group (1.00%). In total, the fatness of carcasses of birds fed with pea seeds (Pe) was lower than the control group, but in terms of the total fat proportion in the carcasses, the WL and NLL groups were characterized by lower values (*p* < 0.001).

### 3.4. Physicochemical Traits

The breast muscles obtained from birds fed with rapeseed meal (RSM) and yellow lupin (YL) were characterized by higher pH_15_ than the control group (SBM), but 24 h after slaughter, a higher pH_24_ was noticed in the RSM group only (Table 7). The lightness (L*) and the redness (a*) of the breast muscles were significantly different than in the SBM group. The RSM group was characterized by higher L* and lower a* (RSM: 50.83 and 2.59, SBM: 47.76 and 3.69, respectively). In values of drip loss, the breast muscles from the YL group were characterized by lower values. This demonstrated a lower ability to retain water (*p* = 0.018). Each experimental group had a significantly lower protein content (*p* < 0.0001) than the SBM group. Fat levels were significantly higher in the YL and Pe groups. At the same time, the water content was higher in the RSM and WL group, and the collagen content was also higher in the WL group, as well as in the group of birds that were fed with pea seeds (*p* < 0.001) (Table 7). As Table 8 shows, only the yellowness (b*) of the leg muscles in the WL and YL groups was significantly higher (6.17 and 6.08, respectively) than in the SBM group (3.69). The water-holding capacity (WHC) of the leg muscles was significantly different in the RSM, WL, and NLL groups in comparison with the SBM group (*p* = 0.002). In the experimental groups, the values of WHC were higher, which means that the leg muscles from the experimental groups lost less water. Similarly, as it was shown in the breast muscle traits, all of the experimental groups were characterized by a lower content of protein. Only the YL and Pe groups had a higher content of fat (*p* < 0.0001). The water content in the leg muscles was significantly different in the RSM, FB (higher content), YL, and Pe (lower content) groups than in the SBM group (73.41%). A higher content of collagen in leg muscles (1.38%) was found in the Pe group only, as compared with the control group (1.02%) (*p* < 0.001) (Table 8). The rest of the physicochemical traits in the breast and leg muscles were not statistically significantly different between the control group, fed with soybean meal (SBM), and the experimental groups, fed with rapeseed meal and various legume seeds (*p* > 0.005).

## 4. Discussion

The effects of diet based on different sources of vegetable protein as an alternative to the popular SBM on the growth performance and quality of carcasses in broiler chickens have been investigated in many studies [31,32,33,34,35,36].

### 4.1. Chemical Composition of Legumes

Pea (Tarchalska), yellow lupin (Mister), and narrow-leaved lupin (Sonet) seeds were also used in duck nutrition in studies by Kuźniacka et al. [18]. The levels of crude protein (CP), ADF, and NDF, as well as starch in pea seeds, were almost the same as in the present research, as was the content of antinutritional substances. The content of CP (416 g/kg·DM) in yellow and narrow-leaved (257 g/kg·DM) lupin seeds was found to be lower in the study by Hejdysz et al. [13], similar to the yellow lupin seeds used in the studies of Rutkowski et al. [9]. Hejdysz et al. [12] showed that raw and extruded faba bean (var. Amulet) seeds contained 326–321 g/kg DM CP, as well as 413–408 g/kg·DM starch and 3.52–2.61 g/kg·DM p-phyt., whereas in our research, the levels of CP and p-phyt. were lower and that of starch higher. Furthermore, the level of oligosaccharides in our research was higher by approximately 30 g/kg·DM. This could have been due to the use of a different faba bean variety (Olga vs. Amulet). Konieczka and Smulikowska [37] studied the nutritional value of different varieties of narrow-leaved (blue) lupin. The CP content was between 269 (var. Regent) and 313 g/kg (var. Tango), while the Sonet variety used in our research had a similar CP level to the Tango variety. In this case, there was an evident relationship between the plant variety and the chemical composition of the seeds. The chemical composition may also have been affected by the processing applied to the seeds, e.g., extrusion [13]. According to Hejdysz et al. [38] faba bean seeds have a similar content of CP and sulfur amino acids (AAs) to soybean meal. However, in our research, differences between FB and SBM seeds were found. Comparing the chemical composition of narrow-leaved lupin seeds in our research (var. Sonet) and the research by Hejdysz et al. [11] (var. Boruta) showed that there were differences in the CP content (by over 80 g/kg·DM), as well as the other ingredients, similarly to the results found in the studies of Kaczmarek et al. [35].

### 4.2. Chickens’ Performance and Meat Quality

The European Broiler Index (EBI) of birds fed with soybean meal in our research was much higher than in all experimental groups (SBM: 507, other groups: 233–348). As Górski et al. [39] reported, the EBI should not be lower than 220, but a value of 190 is also satisfactory.

According to Tuśnio et al. [20], a level of potato protein of less than 10% in broiler chicken diets does not negatively impact the growth performance of broiler chickens. The use of higher potato protein levels (15%) in diets decreases feed intake and broilers’ body weight gain as a result of the increased concentration of solanidine glycoalkaloids and trypsin inhibitor. In our experiment, potato protein was used to even out the protein level in all the experimental diets in amounts no higher than 10%. Therefore, we speculate that the differences in broiler performance found between the experimental groups and SBM group were due to the high contents of ADF and NDF, as well as antinutritional factors included in legume seeds and rapeseed meal.

Other researchers reported [36] that high levels of lupin (30%) in the feed rations for broiler chickens had a negative effect on growth performance compared to birds fed on soybean meal, which was confirmed by our study. Olkowski et al. [33] demonstrated that feeding broiler chickens with seeds of different lupin cultivars reduced FI and BWG. A greater BWG was found only in birds on a diet containing narrow-leaved and white lupin, but it was still lower than the BWG in the birds fed soybean meal. Our study, which compared the effects of protein from different legumes in feed rations, revealed the best growth performance in birds on a diet containing yellow lupin, white lupin, and faba beans, but it was still significantly lower than in the soybean-rich feed group. Similar findings were reported by Meng and Słomiński [40], who compared growth performance in chickens fed complete diets containing soybean meal, rapeseed, and pea.

On the other hand, Laudadio and Tufarelli [41] found that a diet containing peas as a source of protein had a significant effect on the growth performance parameters in broiler chickens, while in our study, this diet was associated with a reduced BWG and an increased FCR. According to Kozłowski et al. [42], growth performance in poultry fed on mixtures containing lupin as a source of protein depends on the lupin cultivar and its level in the diet. A yellow lupin diet in a study by Orda et al. [43] had no negative effect on the growth performance of broiler chickens, while Smulikowska et al. [44] concluded that birds receiving a feed mixture with a 10% content of lupin showed lower growth performance compared to birds fed an SBM diet. Similar values of meat traits were reported by Usayran et al. [45], who compared the performance of birds fed on soybean meal and faba beans. According to Dal Bosco et al. [46], the performance results could be caused by a lower level of essential AAs or the content of tannins, as well as the other antinutritional factors. Significantly lower production rates of chickens fed on rapeseed meal or lupins may be caused by a high content of NDF, ADF, and phytin phosphorus (p-phyt.), which was confirmed in our research. Furthermore, lupins may have a high level of phosphorus in phytic form. Phytic phosphorus is not available for birds [47]. Phytin phosphorus in feed may have a negative effect on the use of protein by birds (increased protein loss) [48]. It also has a negative effect on the digestion and absorption of nutritional components [49]. Experiments carried out by different authors have not demonstrated any negative effects associated with diets containing peas on the traits of chicken carcasses [40,49]. However, in our study, the replacement of SBM with peas significantly reduced the weight of the gutted carcasses with neck. Like in other studies [41,50], we found that dietary peas had no effect on the proportion of breast muscles in the carcasses. On the other hand, studies by Laudadio and Tufarelli [41], and Masoero et al. [50] did not report any negative effects associated with feed mixtures containing peas on the proportion of leg muscles. The abovementioned studies [41,50] found no negative effects associated with dietary peas on the content of subcutaneous fat in carcass. This is consistent with the findings from our study, since there was no significant difference in the content of subcutaneous fat between chickens fed on peas and the control group (SBM). The results regarding abdominal fat were consistent with the findings of Usayran et al. [45], who reported a reduction in abdominal fat in birds fed on diets containing faba beans. Laudadio and Tufarelli [51] analyzed the effect of white lupin on the meat traits of broiler chickens and reported a reduced content of abdominal fat in the carcasses; in our study, however, the content of abdominal fat in chickens fed on white lupin and SBM was similar. Laudadio and Tufarelli [51] also reported no significant differences in the pH_24_ of meat from chickens fed a diet based on white lupin or soybean meal, similarly to our research. The color of meat is one of the most important traits for consumers. Kozłowski et al. [42] reported that the yellowness (b*) of leg muscles was significantly higher in chickens fed on peas, which may suggest a lower level of lipids in the leg muscles. On the other hand, Laudadio and Tufarelli [51] found a significantly lower value of L* (lightness) in the muscles from birds fed on white lupin. Another study [52] on the use of peas as a substitute for soybean meal did not show differences in meat traits or growth performance parameters. Laudadio and Tufarelli [34] concluded that WHC, which affects the loss of water from muscles, is an important parameter in defining the quality of meat. Studies by these authors showed that breast and leg muscles from chickens fed on a pea diet were characterized by a higher WHC (19.64% and 24.22%, respectively) as compared with birds fed on soybean meal (17.23% and 15.21%). Similar results in terms of the composition of muscle tissue were reported by Laudadio and Tufarelli [41]. The water content in meat is around 75%. It is distributed in intra- and extra-microfibrillary spaces. Lower water loss, i.e., a greater ability of muscle fibers to accumulate it, testifies to a better technological suitability of meat for further processing [53]. A reduced content of abdominal fat in carcasses, higher values of L* and a*, and a better water-holding capacity of meat may indicate higher protein content in the muscles and a higher concentration of fat-soluble pigments. Wilhelm et al. [54] reported that meat with L* = 52.00 could be considered normal breast muscles, and L* = 57.37 was found in breast muscles with PSE (pale, soft, exudative). Thus, it may be concluded that the meat in our research was not PSE. In the study of Kuźniacka [15], it was concluded that use of faba bean seeds could be proposed in broilers’ diets. However, the results of physicochemical parameters were a little bit different than in our study. This could be due to the different levels of faba beans that were used in the two studies.

## 5. Conclusions

This study demonstrated that a diet containing faba beans with the addition of potato protein and brewers’ yeast had no negative effect on the analyzed traits, excluding growth performance. The quality of the meat from chickens fed on faba beans as a high-protein component in combination with potato protein and brewers’ yeast was like that in birds fed on mixtures formulated with soybean meal. Furthermore, the use of lupins had no negative effect on most of the carcass traits of the chickens. In addition, faba beans are characterized by the low content of antinutrients and could be proposed as an alternative for soybean meal in broiler chicken nutrition. The results of meat quality from broiler chickens fed with faba beans had similar values as the birds fed with soybean meal. The European Broiler Index values of birds fed with rapeseed meal and legume seeds were lower than from birds fed with soybean meal, but as can be seen in the sources, the values achieved in our research were still at a good level.

## Figures and Tables

**Table 1:** Chemical composition of legume seeds (g/kg dry matter).

Component	Pea (Tarchalska)	Faba Bean (Olga)	Soybean Meal	Rapeseed Meal	Yellow Lupin (Mister)	Narrow-Leaved Lupin (Sonet)	White Lupin (Butan)
Crude protein	204	292	451	341	425	303	341
ADF	91	117	77	191	194	205	210
NDF	165	188	119	251	252	255	187
Crude fat	10	10	15	31	35	49	94
Starch	590	480	nd^2^	nd^2^	nd^2^	nd^2^	nd^2^
*Essential amino acid (g/16g·N)*							
Arginine	12.2	9.77	7.51	6.01	15.1	11.6	10.9
Cysteine	0.86	0.98	1.42	2.64	1.91	0.82	1.17
Glycine	4.11	4.00	4.20	5.37	4.28	4.05	4.11
Histidine	2.73	2.78	2.50	2.69	3.05	2.69	2.63
Isoleucine	3.94	3.85	4.63	3.38	4.30	4.01	4.39
Leucine	7.06	7.08	7.18	7.18	8.85	6.89	7.55
Lysine	7.91	6.67	5.51	5.19	6.66	5.45	5.59
Methionine	0.57	0.89	1.19	2.09	0.53	0.36	0.54
Phenylalanine	4.59	4.09	4.86	4.22	4.42	4.02	4.18
Threonine	3.50	3.46	3.48	4.58	3.66	3.41	4.27
Valine	4.85	4.35	4.17	5.0	4.04	3.92	4.31
*Non-essential amino acid (g/16g·N)*							
Alanine	4.11	3.89	4.22	4.52	3.54	3.37	3.45
Asparagine acid	12.2	10.2	11.0	7.91	11.2	9.98	10.9
Glutamine acid	15.8	14.9	18.0	15.8	25.2	19.5	18.2
Proline	3.82	4.01	5.21	6.23	3.61	3.71	3.16
Serine	4.53	4.41	5.13	4.47	5.51	4.94	4.91
Tyrosine	2.83	2.92	3.48	3.12	3.09	3.24	4.42
*Antinutrients*							
Total alkaloids	-	-	-	-	0.0009	0.0017	0.0019
Oligosaccharides (RTO)^3^	62.91	53.61	46.2	20.4	134.2	58.06	76.2
Raffinose	8.36	3.15	7.70	3.40	10.2	7.07	7.90
Stachyose	27.13	14.02	35.5	17.0	82.8	37.23	56.9
Verbascose	27.42	36.44	3.00	-	41.2	13.76	11.3
P—phyt.	1.8	1.7	4.2	7.9	5.0	2.3	2.1
Tannin	0.02	0.06	-	-	-	-	-

^1^ Each value represents mean of four replicates; nd—not determined; ^2^ Starch was not determined because the content of this compound in lupines is very low (around 1%) and the reliability of results is uncertain; ^3^ RTO—raffinose-type oligosaccharides.

**Table 2:** Composition of feed mixtures (%) for broiler chickens—starter.

Components—Starter	1	II	III	IV	V	VI	VII
Corn	58.73	53.33	53.28	55.24	51.56	51.34	51.17
Soybean meal	25	-	-	-	-	-	-
Rapeseed meal	-	25	-	-	-	-	-
White lupin, cv. Butan	-	-	25	-	-	-	-
Yellow lupin, cv. Mister	-	-	-	25	-	-	-
Narrow-leaved lupin, cv. Sonet	-	-	-	-	25	-	-
Pea, cv. Tarchalska	-	-	-	-	-	25	-
Faba bean, cv. Olga	-	-	-	-	-	-	25
Potato protein	3.8	7.4	8.1	5.80	8.00	10.80	9.50
Brewers’ yeast	4	4	4	5	6	6	6.00
Soybean oil	3.85	6.2	5	4	4.9	2.4	3.74
Premix 1% ^a^	1	1	1	1	1	1	1
Monocalcium phosphate	1.5	1.35	1.78	1.59	1.55	1.58	1.80
Fodder chalk	0.62	0.35	0.48	0.63	0.58	0.70	0.56
Fodder salt	0.07	0.05	0.05	0.05	0.05	0.08	0.09
NaHCO_3_	0.53	0.53	0.53	0.53	0.53	0.53	0.51
L-lysine	0.37	0.49	0.40	0.48	0.41	0.26	0.25
DL-methionine	0.21	0.15	0.23	0.26	0.22	0.18	0.22
L-threonine	0.13	0.08	0.06	0.16	0.07	0.04	0.07
L-valine	0.19	0.07	0.09	0.26	0.13	0.09	0.09
*Calculated nutritional value of experimental diets (%)*							
ME, kcal/kg	3048	3056	3047	3054	3050	3055	3055
Crude protein	21.95	21.94	21.95	21.98	21.94	21.90	21.94
Calcium	0.96	0.96	0.96	0.96	0.96	0.96	0.96
Ether extract	6.78	9.13	9.46	7.70	8.57	5.10	6.36
Starch	37.3	33.4	33.4	34.7	32.5	42.9	41.6
Phosphorus	0.48	0.48	0.48	0.48	0.48	0.48	0.48
dig. Lysine	1.28	1.28	1.28	1.28	1.28	1.28	1.28
dig. Methionine	0.51	0.51	0.51	0.51	0.51	0.51	0.51
dig. Threonine	0.86	0.86	0.86	0.86	0.86	0.86	0.86
Valine	1.1	1.1	1.1	1.1	1.1	1.1	1.10
Sodium	0.20	0.19	0.19	0.20	0.20	0.20	0.20
Chloride	0.18	0.18	0.18	0.18	0.18	0.18	0.18
NDF	9.40	13.1	11.2	11.9	12.2	9.90	11.2
ADF	3.44	6.41	6.56	6.33	7.21	3.77	4.60

^a^ Vitamin–mineral premix provided per kg diet: Mn, 55 mg; Zn, 50 mg; Fe, 80 mg; Cu, 5 mg; Se, 0.1 mg; I, 0.36 mg; Na, 1.6 g, retinol, 2.48 mg; cholecalciferol 25 μg; DL-α-tocopherol, 60 mg; cyanocobalamin, 0.012 mg; menadione sodium bisulphite, 1.1 mg; niacin, 53 mg; choline chloride, 1020 mg; folic acid, 0.75 mg; biotin, 0.25 mg; riboflavin, 5.5 mg.

**Table 3:** Composition of feed mixtures (%) for broiler chickens—grower.

Components—Grower	1	II	III	IV	V	VI	VII
Corn	58.78	52.81	52.67	54.75	50.96	50.84	50.75
SBM	25	-	-	-	-	-	-
RSM	-	25	-	-	-	-	-
White lupin, cv. Butan	-	-	25	-	-	-	-
Yellow lupin, cv. Mister	-	-	-	25	-	-	-
Narrow-leaved lupin, cv. Sonet	-	-	-	-	25	-	-
Pea, cv. Tarchalska	-	-	-	-	-	25	-
Faba bean, cv. Olga	-	-	-	-	-	-	25
Potato protein	4.40	7.10	7.80	5.50	7.70	10.50	9.20
Brewers’ yeast	2.50	4	4	5	6	6	6
Soybean oil	5.40	7.70	6.60	5.50	6.50	3.90	5.20
Premix 1% ^a^	1	1	1	1	1	1	1
Monocalcium phosphate	1.35	1.10	1.55	1.35	1.30	1.35	1.56
Fodder chalk	0.48	0.24	0.34	0.50	0.46	0.57	0.44
Fodder salt	0.13	0.08	0.10	0.11	0.12	0.15	0.15
NaHCO_3_	0.48	0.51	0.48	0.46	0.46	0.42	0.41
L-lysine	0.21	0.35	0.26	0.34	0.26	0.12	0.10
DL-methionine	0.17	0.11	0.19	0.23	0.19	0.15	0.18
L-threonine	0.04	-	-	0.08	-	-	-
L-valine	0.06	-	0.01	0.18	0.05	-	0.01
*Calculated nutritional value of experimental diets (%)*							
ME, kcal/kg	3156	3152	3150	3151	3152	3153	3150
Crude protein	21.56	21.49	21.50	21.55	21.49	21.47	21.50
Ether extract	8.29	10.6	11.0	9.18	10.2	6.57	7.8
Starch	37.2	33.1	33.0	34.4	32.1	42.6	41.3
Calcium	0.87	0.87	0.87	0.87	0.87	0.87	0.87
Phosphorus	0.43	0.43	0.43	0.43	0.43	0.43	0.43
dig. Lysine	1.15	1.15	1.15	1.15	1.15	1.15	1.15
dig. Methionine	0.47	0.47	0.47	0.47	0.47	0.47	0.47
dig. Threonine	0.77	0.77	0.79	0.77	0.77	0.81	0.77
Valine	1	1.01	1	1	1	1	1
Sodium	0.20	0.20	0.20	0.20	0.20	0.20	0.20
Chloride	0.19	0.19	0.18	0.19	0.19	0.19	0.19
NDF	9.50	13.0	11.0	11.8	12.1	9.80	11.1
ADF	3.45	6.39	6.53	6.32	7.19	3.75	4.59

^a^ Vitamin–mineral premix provided per kg diet: Mn, 55 mg; Zn, 50 mg; Fe, 80 mg; Cu, 5 mg; Se, 0.1 mg; I, 0.36 mg; Na, 1.6 g, retinol, 2.48 mg; cholecalciferol 25 μg; DL-α-tocopherol, 60 mg; cyanocobalamin, 0.012 mg; menadione sodium bisulphite, 1.1 mg; niacin, 53 mg; choline chloride, 1020 mg; folic acid, 0.75 mg; biotin, 0.25 mg; riboflavin, 5.5 mg.

**Table 4:** Growth performance parameters (means) of broiler chickens during the 6 week rearing period.

Source of Vegetable Protein ^1^	BWG (g)			FI (g)			FCR (kg/kg)			EBI
	0–14 d	15–42 d	0–42 d	0–14 d	1–42 d	0–42 d	0–14 d	15–42 d	0–42 d	
SBM	331	2337	2599	479	3347	3967	1.45	1.52	1.51	468
RSM	231 *	1655 *	1879 *	427	3625	4100	1.91 *	2.22 *	2.18 *	252 *
WL	230 *	2060 *	2216 *	500	3902 *	4401 *	2.18 *	1.91 *	1.93 *	348 *
YL	237 *	1987 *	2215 *	451	3872 *	4335 *	2.12 *	1.95 *	1.96 *	331 *
NLL	208 *	1850 *	2058 *	478	3931 *	4409 *	2.31 *	2.13 *	2.15 *	281 *
Pe	201 *	1587 *	1782 *	425 *	3508	3933	2.15 *	2.25 *	2.24 *	233 *
FB	216 *	1892 *	2107 *	461	3634	4095	2.10 *	1.92 *	1.95 *	316 *
SEM	7.00	36.0	37.0	6.00	41.0	36.0	0.04	0.03	0.03	12.30
*p*	<0.001	<0.001	<0.001	0.003	<0.001	<0.001	<0.001	<0.001	<0.001	<0.001

* Means in a column differ from control treatment, *p*-value < 0.05; ^1^ SBM: soybean meal; RSM: rapeseed meal; WL: white lupin; YL: yellow lupin; NLL: narrow-leaved lupin; Pe: pea; FB: faba bean; SEM: standard error of the mean; BWG: body weight gain (g); FI: feed intake (g); FCR: feed conversion ratio (kg/kg); EBI: European Broiler Index.

**Table 5:** Meat traits of 6 week old broiler chickens (means).

Source of Vegetable Protein ^1^	Pre-Slaughter Body Weight (g)	Weight of Gutted Carcass with Neck (g)	Dressing Percentage (%)	Weight and Proportion in Carcass				Weight of Offal (g)	Carcass Remains (g)
				Neck		Wings			
				g	%	g	%		
SBM	2370	1680	70.6	36.1	2.15	171	10.2	95.8	506
RSM	2032 *	1397	68.7	27.6	1.99	151	10.8	98.8	464
WL	2270	1536 *	67.6	27.2	1.80	167	10.9	98.5	506
YL	2206	1495 *	67.8	29.9	2.01	160	10.8	105	520
NLL	2213 *	1348 *	66.5 *	65.7 *	4.47 *	159	10.8	100	443
Pe	2071 *	1405 *	67.8	27.7	1.98	157	11.1	106	460
FB	2197	1515	68.9	35.6	2.37	161	10.6	100	456
SEM	143	27.2	0.24	1.92	0.13	2.3	0.11	1.40	8.8
*p*	<0.001	0.019	<0.001	<0.001	<0.001	0.323	0.558	0.421	0.105

* Means in a column differ from control treatment, *p*-value < 0.05; ^1^ SBM: soybean meal; RSM: rapeseed meal; WL: white lupin; YL: yellow lupin; NLL: narrow-leaved lupin; Pe: pea; FB: faba bean; SEM: standard error of the mean.

**Table 6:** Content of muscles and fat in 6 week old broiler chickens (means).

Source of Vegetable Protein ^1^	Weight and Proportion in Carcass											
	Muscles						Skin with Subcutaneous Fat		Abdominal Fat		Total Fat	
	Breast		Legs		Total							
	g	%	g	%	g	%	g	%	g	%	g	%
SBM	440	26.1	351	20.8	791	47.0	162	9.65	16.7	1.00	17	10.6
RSM	329 *	23.5	278 *	19.9 *	608 *	43.4 *	125 *	8.93	21.8	1.55 *	147 *	10.5
WL	368 *	23.8	334	21.7	702	45.5	122 *	7.87 *	13.8	0.93	144 *	8.71 *
YL	307 *	20.5 *	336	22.4	643 *	42.9 *	131 *	8.72	12.9	0.85	143 *	9.49
NLL	378	25.7	299 *	20.4 *	677 *	46.1	116 *	7.87 *	12.5	0.85	127 *	8.64 *
Pe	329 *	23.5	281 *	19.9 *	610 *	43.4 *	138	9.78	15.0	1.04	151	10.7
FB	395	26.1	333	21.9	728	48.1	127 *	8.34	8.90 *	0.59	135 *	8.93
SEM	8.27	0.39	5.70	0.228	12.50	0.463	3.20	0.171	0.863	0.059	3.27	0.193
*p*	<0.001	<0.001	<0.001	0.003	<0.001	0.008	0.001	0.007	0.001	<0.001	<0.001	<0.001

* Means in a column differ from control treatment, *p*-value < 0.05; ^1^ SBM: soybean meal; RSM: rapeseed meal; WL: white lupin; YL: yellow lupin; NLL: narrow-leaved lupin; Pe: pea; FB: faba bean; SEM: standard error of the mean.

**Table 7:** Physicochemical parameters of breast muscles from 6 week old broiler chickens (means).

Source of Vegetable Protein ^1^	pH_15_	pH_24_	Color ^2^			Water-Holding Capacity (%)	Drip Loss (%)	Protein (%)	Fat (%)	Water (%)	Collagen (%)
			L*	a*	b*						
SBM	6.02	5.96	47.8	3.69	5.03	63.1	99.6	24.0	1.68	74.1	0.63
RSM	6.26 *	6.00 *	46.5	3.93	4.41	64.7	99.6	23.0 *	1.85	74.8 *	0.66
WL	5.97	5.94	50.8 *	2.59 *	6.07	65.7	99.4	23.4 *	1.84	74.5 *	0.73 *
YL	5.89 *	5.92	50.4	3.24	6.13	64.7	98.9 *	22.8 *	2.37 *	74.0	0.61
NLL	5.99	5.95	49.1	3.27	5.43	66.1	99.1	23.7 *	1.66	73.8	0.73
Pe	5.97	5.95	49.1	3.40	3.69	65.1	99.3	23.2 *	1.88 *	74.2	0.83 *
FB	6.06	5.97	49.7	2.86	4.76	64.5	99.3	23.6	1.84	74.2	0.63
SEM	0.02	0.01	0.34	0.113	0.212	0.56	0.06	0.05	0.03	0.05	0.021
*p*	<0.001	0.001	0.004	0.027	0.130	0.875	0.018	<0.001	<0.001	<0.001	0.014

* Means in a column differ from control treatment, *p*-value < 0.05; ^1^ SBM: soybean meal; RSM: rapeseed meal; WL: white lupin; YL: yellow lupin; NLL: narrow-leaved lupin; Pe: pea; FB: faba bean; SEM: standard error of the mean; ^2^ L*: lightness; a*: redness; b*: yellowness.

**Table 8:** Physicochemical parameters of leg muscles from 6 week old broiler chickens (means).

Source of Vegetable Protein ^1^	Color ^2^			Water-Holding Capacity (%)	Drip Loss (%)	Protein (%)	Fat (%)	Water (%)	Collagen (%)
	L*	a*	b*						
SBM	49.2	6.18	3.69	59.5	98.6	20.3	5.69	73.4	1.02
RSM	48.1	5.46	4.36	66.7 *	96.0	19.4 *	5.89	74.0 *	1.11
WL	49.6	4.58	6.17 *	66.2 *	99.5	19.6 *	5.87	73.5	1.04
YL	49.8	5.21	6.08 *	59.8	99.3	19.2 *	7.60 *	71.9 *	1.11
NLL	48.3	5.58	4.78	65.9 *	99.5	19.9 *	5.86	73.2	1.10
Pe	49.6	4.73	4.50	62.7	99.5	19.2 *	7.21 *	72.6 *	1.38 *
FB	48.6	5.34	4.80	63.5	99.4	19.8 *	5.42	74.1 *	1.04
SEM	0.34	0.146	0.238	0.61	0.478	0.05	0.10	0.09	0.02
*p*	0.754	0.065	0.045	0.002	0.444	<0.001	<0.001	<0.001	<0.001

* Means in a column differ from control treatment, *p*-value < 0.05; ^1^ SBM: soybean meal; RSM: rapeseed meal; WL: white lupin; YL: yellow lupin; NLL: narrow-leaved lupin; Pe: pea; FB: faba bean; SEM: standard error of the mean; ^2^ L*: lightness; a*: redness; b*: yellowness.
